# Method to determine the root canal spatial geometry using an algorithm of the e-Vol DX CBCT software

**DOI:** 10.1590/0103-6440202305661

**Published:** 2023-12-22

**Authors:** Carlos Estrela, Mike Reis Bueno, Giampiero Rossi-Fedele, Daniel Almeida Decurcio, Orlando Aguirre Guedes, Manoel Damião Sousa-Neto, Cyntia Rodrigues de Araújo Estrela

**Affiliations:** 1 Professor of Endodontics, School of Dentistry, Federal University of Goiás, Goiânia, Brazil; 2 CROIF Diagnostic Imaging Center, Cuiabá, Brazil; 3 Professor of Endodontics University of Adelaide, Dental School, Department of Endodontics, Adelaide, Australia.; 4 Professor of Oral Biology, School of Dentistry, Evangelical University of Goiás, GO, Brazil; 5 Professor of Endodontics, School of Dentistry, University of São Paulo, Ribeirão Preto, SP, Brazil

**Keywords:** Anatomy, Cone-beam computed tomography, Geometry, Root canal preparation, Software

## Abstract

This study evaluated a method to determine the spatial geometry of root canal preparation (RCP) using navigation dynamics and a specific algorithm from a new CBCT software (e-Vol DX). CBCT scans of 168 root canals of mandibular molars were acquired before and after RCP, using nickel-titanium (NiTi) instruments (ProTaper Next, BioRace, Reciproc Blue, and WaveOne Gold). The spatial geometry of the root canals and the operative risk of disproportional wear of dentinal walls after RCP were evaluated using a new CBCT software. A 3-point scoring system was used after the measuring of cementum/dentin thickness before and after RCP in all root thirds. The root thirds were distributed into three parts of similar sizes, and the scores were categorized at three levels: 1. mild risk (1/3), 2. moderate risk (2/3), 3. severe risk (3/3). These levels were proposed according to the risk of creating disproportionate shapes, thin walls, or perforations. The data were analyzed statistically by Fischer’s exact test (α = 5%). There were no significant differences in operative risk among the NiTi engine-driven systems, for the distal or mesial walls of all the root canal thirds (p>0.05). The spatial geometry method to assess operative risk allows clinical planning for a predictable enlargement of the root canal in all root thirds. Based on using a map-reading strategy on root canals in CBCT scans, NiTi engine-driven instruments did not present an increased operative risk during RCPs.

## Resumo

Este estudo avaliou um método para determinar a geometria espacial do preparo do canal radicular (PCR) usando uma dinâmica de navegação e um algoritmo específico do software e-Vol DX. Imagens de tomografia computadorizada de feixe cônico (TCFC) de 168 molares inferiores foram adquiridas antes e depois do PCR, usando instrumentos de níquel-titânio (NiTi) (ProTaper Next, BioRace, Reciproc Blue e WaveOne Gold). A geometria espacial dos canais radiculares e o risco operatório de desgaste desproporcional das paredes dentinárias após o preparo foram avaliados usando o software de TCFC e-Vol DX. Um sistema de Score de 3 pontos foi usado após a mensuração da espessura cemento/dentina antes e depois do PCR, em toda extensão dos canais radiculares. Os terços radiculares foram distribuídos em três partes de tamanhos semelhantes, e os Scores foram categorizados em três níveis: 1. risco leve (1/3), 2. risco moderado (2/3), 3. risco severo (3/3). Esses níveis foram propostos de acordo com o risco de criar formas desproporcionais, paredes finas ou perfurações radiculares. Os dados foram analisados estatisticamente pelo teste exato de Fischer ((=5%). Não houve diferenças significativas no risco operatório entre os sistemas de NiTi acionados a motor, para as paredes distal ou mesial em todos os terços do canal radicular (p>0,05). O método de geometria espacial para avaliar o risco operatório permite o planejamento clínico para um alargamento previsível do canal radicular em todos os terços radiculares. Com base no uso de uma estratégia de navegação dinâmica de canais radiculares de molares inferiores analisados em imagens de TCFC, os instrumentos de NiTi acionados estudados não apresentaram um aumento do risco operatório durante o preparo dos canais radiculares.

## Introduction

The determining factors for successful root canal treatment are based on the fundamentals of correct diagnosis, emptying, enlargement (shape), sanitization, and sealing ^1^ ^,^ ^2^. Concerns regarding microbial biofilm remain ongoing, and the action of an efficient biocidal strategy is required to destroy it ^1^. The incorporation of nickel-titanium (NiTi) engine-driven instruments in the clinical routines influences RCP quality, particularly due to the essential characteristics of the alloy, including elasticity and flexibility ^3^ ^,^ ^4^ ^,^ ^5^. These engine-driven systems have reduced canal transportation compared to stainless-steel files ^4^ ^,^ ^5^ ^,^ ^6^. Root canal preparation (RCP) must be performed within safety principles, avoiding unnecessary wear and tear that could jeopardize clinical success ^3^ ^,^ ^7^. Iatrogenic procedural errors (such as stepping, perforation, root canal decentralization, and apical transportation) constitute risk factors for RCT failure ^1^.

The history of Endodontics has recorded progressive advances to overcome the difficulties in visualizing the final shape after RCP ^8^ ^,^ ^9^ ^,^ ^10^ ^,^ ^11^ ^,^ ^12^ ^,^ ^13^ ^,^ ^14^ ^,^ ^15^ ^,^ ^16^ ^,^ ^17^. The anatomic complexity of the root canal visualized in three dimensions, and its variations ^11^ ^,^ ^16^, have shown the challenges and limitations of using two-dimensional imaging exams. New technologies have a positive impact on shape quality after RCP. Design and innovation in the manufacture of flexible instruments ^3^, as well as correct planning and clinical management using sophisticated cone-beam computed tomography (CBCT) software ^2^ have increased the anticipated standard of RCP.

 The root canal shape before and after the action of different instruments has been investigated through different strategies including periapical radiographs ^14^, acrylic-resin blocks ^9^, simulated root canals ^17^, and graphics computer analysis ^8^. Micro-computed tomography (MCT) has been used in several studies ^12^ ^,^ ^13^ ^,^ ^15^ showing the area and volume changes of the root canal shape, apical transportation, and root canal geometry. The inclusion of CBCT in endodontics provides clinical benefits and aids in diagnosis, treatment planning, and decision-making ^2^ ^,^ ^18^ ^,^ ^19^ ^,^ ^20^. The centralization ability and root canal transportation during the RCP with NiTi rotary and stainless-steel instruments have been analyzed by using CT ^21^, and CBCT ^22^.

The manufacturers of endodontic instruments have introduced new technologies aiming at improving cleaning and shaping ^7^ ^,^ ^10^. Innovations have been introduced in the cinematics of instruments, and thermal treatments of NiTi alloys (Max-Wire technology - M-Wire and Controlled Memory - CM) ^23^. Parallel to advances with endodontic instruments, a recent CBCT software (e-Vol DX)^2^ has allowed high-quality CBCT images, visualization of complex anatomical structures ^24^ ^,^ ^25^ ^,^ ^26^, precise identification of occult lesions ^19^, reduction of artifacts ^27^, a replication of positions in the three-dimension (3D) mode in multiplanar reconstruction (MPR) ^28^, filter for the measurements ^28^ ^,^ ^29^, the use of cinematic rendering tool ^30^, and standardize image adjustments to analyze CBCT volumes from different sources, and various other filters ^2^.

The anatomical complexity of the root canal causes challenges to obtaining appropriate access and enlargement of the entirety of the root canal walls by endodontic instruments, which can interfere with the quality of microbial decontamination. Thus, new strategies and methodologies for analyzing the spatial geometry of RCP, with potential applications in clinical practice must be assessed. This study evaluated a CBCT imaging method to determine a predictable enlargement of the root canal in all root thirds, based on the spatial geometry of root canal preparation (RCP).

## Methodology

### Sample Selection

Raw (primary) data was collected originally from a previous study ^31^ on the thickness of cementum-dentin in the danger zone of only one operative risk checkpoint. Based on these primary data and applying a dynamic navigation model using an algorithm of CBCT software (e-Vol DX) and operative risk criteria for the entire length of the root canal it can establish new parameters of a method to determine the remaining dentin thickness and the spatial geometry.

This study was approved by the Institutional Ethics Committee (#06486919.0.0000.5083).

The sample size was calculated based on a power analysis using G*Power software version 3.1.2 (Heinrich Heine Universitat, Düsseldorf, Germany) at an alpha error probability of 0.05 and power of 0.95 (effect size = 0.4). The power analysis showed that a total of 112 samples would be necessary. In the present study, 168 samples were used.

First and second human mandibular molars, extracted for different reasons, were assessed to obtain 168 root canals (84 teeth, mesiobuccal, and mesiolingual) and stored in 0.1% thymol until used. The exclusion criteria were history of root canal treatment, orthodontic treatment, posts, crown, internal or external root resorption, calcifications, incomplete rhizogenesis, and developmental disorder. In the inclusion criteria for the mandibular molars, the root curvature should include a radius greater than 4 mm shorter than 8 mm ^29^, and a length of at least 20 mm. High-resolution CBCT images were used for sample selection.

### Imaging Methods

CBCT scans were acquired before and after RCP using a PreXion 3D scanner (Prexion 3d Inc, San Mateo, CA, USA) according to the following high-resolution protocol: voxel size of 0.100 mm, field of view of 5.6cm X 5.2cm, exposure time of 37 seconds, tube voltage of 90 kV, and tube current of 4 mA. The DICOM files were examined using e-Vol DX software (CDT Software, São José dos Campos, SP, Brazil) installed on a PC workstation equipped with an Intel i7-7700K processor, 4.20 GHz (Intel Corp, Santa Clara, CA, USA), an NVIDIA GeForce GTX 1070 video card (NVIDIA Corporation, Santa Clara, CA, USA), Dell P2719H monitor at a resolution of 1920 X 1080 pixels (Dell Technologies Inc, Round Rock, TX, USA), and Windows 10 Pro (Microsoft, Redmond, WA, USA).

### Root canal preparation

The teeth were removed from the thymol, washed in running water, dried, and immersed in 5% sodium hypochlorite for 30 min to remove all external organic tissues. The access cavity was prepared using a high-speed handpiece under refrigeration, with round diamond burs (#1013, #1014) and an Endo-Z bur (Dentsply/Maillefer, Ballaigues, Switzerland). Mesiobuccal and mesiolingual root canals were explored and emptied using stainless steel #10 and #15 K-files (Dentsply/Maillefer, Ballaigues, Switzerland). The coronal third was prepared using instruments from the system under study. The working length was determined using a #15 K-file by visualization of the file tip through the apical foramen, 1 mm short of this length. All canals were irrigated with 2.5% sodium hypochlorite, using 10 mL of irrigant at each instrument change, applied with a Navitip Tip irrigation (0.30 mm, Ultradent, South Jordan, UT, USA), at 2 mm short of the working length. The patency was confirmed with a #15 K file. The canals were dried with paper points and irrigated with 5 mL of 17% EDTA, which was left in the canal for 3 minutes to remove the smear layer. The 168 canals (mesiobuccal and mesiolingual) were prepared using a sequence of instruments until the diameter corresponding to each NiTi engine-driven instrument was achieved: G1 (n=42) - ProTaper Next (Dentsply/Maillefer, Ballaigues, Switzerland) (X1, #17.04; X2, #25.06; X3, #30.07; X4, #40.06) used according to the manufacturer’s instructions, at 300 rpm and 2.5 N/cm^2^ torque; G2 (n=42) - BioRace (FKG Dentaire, Switzerland) (BR0, #25.08; BR1, #15.05; BR2, #25/0.04; BR3, #25.06; BR4, #35.04; BR5, #40.04) used according to the manufacturer’s instructions at 600 rpm and 1.5 N torque, and with an X-Smart Plus Dentsply/Maillefer, Ballaigues, Switzerland) motor; G3 (n=42) - Reciproc Blue (VDW, Germany) (R25, #25/variable taper followed by R40, #40/variable taper) in an X-Smart Plus motor according to the manufacturer’s instructions for speed and torque using the Reciproc ALL mode; G4 (n=42) - WaveOne Gold (Dentsply/Maillefer, Ballaigues, Switzerland) (Small, #20/variable taper; Primary, #25/ variable taper, Medium, #35/ variable taper) used according to the manufacturer’s instructions for speed and torque using the WaveOne Gold^®^ mode. The irrigation process followed the same procedures as described above. The instruments in all groups were replaced after the RCP of three canals. Final CBCT scans were obtained after the completion of RCP, to evaluate the spatial geometry of the root canal before and after RCP.

### Method to determine spatial geometry

**Figure 1:**
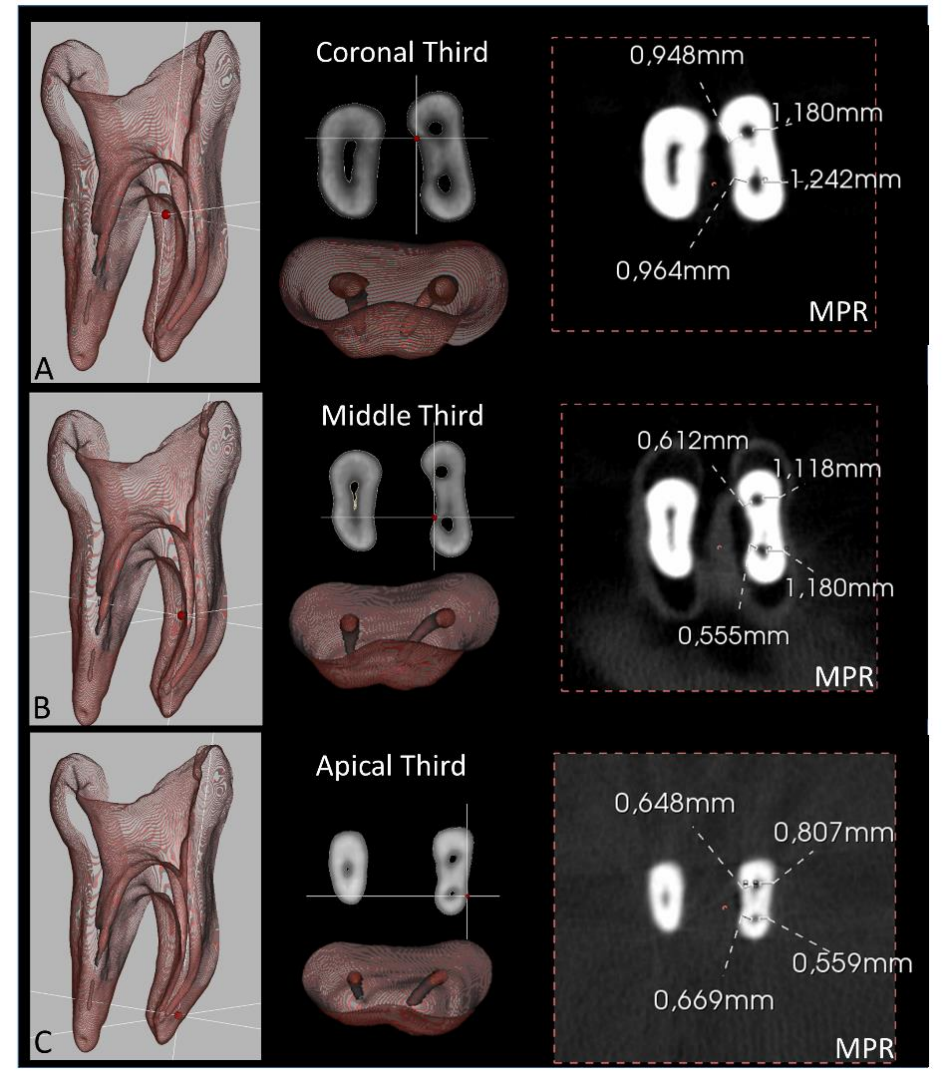
MPR in axial plane showing measurements and synchronized 3D volumetric reconstruction in coronal (A), middle (B), and apical thirds (C). A specific algorithm (pulp cavity) of CBCT e-Vol DX software was developed being able to suppress the cementum/dentin structures and show the relationship between the pulp cavity with the periodontal ligament, in addition, to viewing the volume, the final shape, and taper of the prepared root canal.

The method used to determine the spatial geometry of root canals was based on the operative risk of disproportional wear of dentinal walls after RCP and was evaluated by using a specific algorithm of a new CBCT software (e-Vol DX). Initially, the root canals were aligned axially, and the sagittal and coronal planes were used to keep the long axis of the tooth parallel to the base to avoid parallax error. Next, the anatomical diameter of root canals and cementum-dentin thickness (in buccal-lingual/palatal and mesiodistal directions) were measured on the CBCT scans using a specific algorithm (pulp cavity) of the e-Vol DX CBCT software (2, 28). The measurements were taken along the root canal (coronal, middle, and apical thirds) of the lower cementum-dentin thickness (i.e., coronal third - 3 mm from the furcation; medium third - 6 mm from the furcation; apical third - 1 mm short from the apical foramen) (Figure 1).

The method of measurement of root canal anatomic dimensions ^28^ consisted of establishing the correct position to be measured in 2D mode (multiplanar reformation, MPR) in CBCT scans, defining a point on the border of the anatomical structure, and then adjusting the intermediate position in the grayscale of the CBCT image. Thin slices of 0.10 mm were obtained from the 3D images using the measurement algorithm, and the edge of the anatomic surface was defined on the axial plane. Positions in the 3D model were replicated in multiplanar CBCT image reconstruction, and the correct position was determined using a guide. The 3D density was adjusted to be the same dimension as the 2D image, and dimensions were subsequently calibrated until 3D, and 2D modes were coincident. Thus, the intermediate position in the grayscale was checked on the CBCT scans. After one side was completed, the guide was moved to the other side, following the same steps. The mark position was defined on the other edge using the 2D model as a reference. The measurements in the two edges of the root canal were then made. The dynamic navigation in CBCT scans was performed in axial, sagittal, and coronal sections of 0.1 x 0.1 mm, from the orifice of the coronal chamber to the apical foramen and from the root apex to the coronal region.

**Figure 2:**
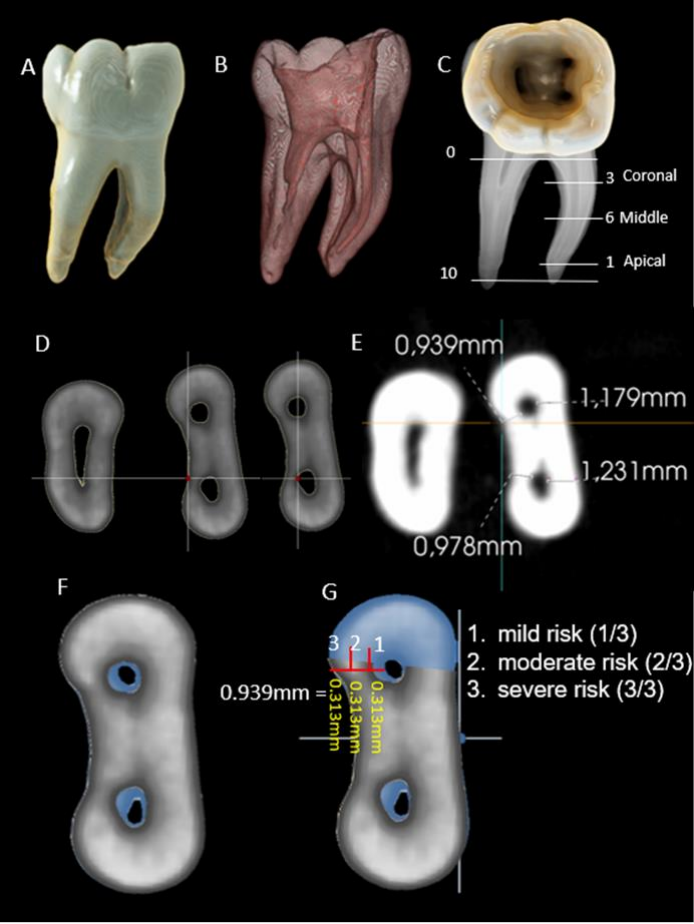
CBCT scans show the volumetric reconstruction and the regions selected to be evaluated (A-E). The measurements were taken along the root canal (coronal, middle, and apical thirds) of the lower cementum-dentin thickness (i.e., coronal third - 3 mm from the furcation; medium third - 6 mm from the furcation; apical third - 1 mm short from the apical foramen)(C-E). Measurement of cementum/dentin thickness before (axial slice in blue) and after (axial slice in gray) RCP in all root thirds, distributed into three (3/3)

Based on this method, the measurement of the apical anatomical diameter and cementum-dentin thickness in any third of the root canal was evaluated before RCP, using dynamic navigation through the CBCT scans (in MPR and 3D volumetric reconstruction with e-Vol DX Software) (Figure 1). The criteria to determine the root canal spatial geometric was based on obtaining the measures of cementum/dentin thickness before and after RCP in all root lengths (extension), distributed into three parts of similar thicknesses, and categorized into three levels: 1. mild risk (1/3), 2. moderate risk (2/3), 3. severe risk (3/3). These levels were established according to the operative risk of creating disproportional shapes, thin walls, or perforations after RCP (Figure 2). The category of relative risk was considered to be cementum/dentin thickness up to 0.5 mm, depending on the tooth, root canal shape, and the third analysis. In the analysis of the apical third, it was considered that cementum/dentin thickness up to 0.5 mm constituted a moderate risk, and those smaller than 0.5 mm were considered to be a severe risk.

After the conclusion of RCP, the volumetric reconstructions of the images (in 3D mode) were superimposed on those initial images obtained before RCP, to determine the level of operative risk in altering the root canal spatial geometry (Figure 2). A specific 3D volume analysis algorithm was also developed to assess the taper of the entire volume of the RCP. A unique tool was developed in the e-Vol DX software to synchronize the CBCT scan in 2D mode (multiplanar reformations, MPR) to 3D mode (volumetric reconstruction, volume rendering), offering visualization of the 3D slice. In this way, the operative risks established by measurement of the remnant thickness of root walls prepared with different instruments may be overlapped and analyzed in 3D reconstructions. A recording system with image alignment in the same 3D position (3D overlap of one body on another) was applied. The spatial geometry of the root canal was characterized using an analysis of shape, size, and position (Figure 2).

This method uses root canal spatial geometry to identify operative risk after RCP and ultimately assess possible root damage (creation of a thin root wall or perforation). This damage is often associated with the distal wall in the coronal third, the furcation area (danger zone), or the mesial wall in the apical third (common area of apical transportation). The RCP must respect the features of the internal canal anatomy, and at the same time may be defined by the characteristics of the instrument used. Establishing a guide for analyzing the root canal anatomy before treatment is essential and requires taking into consideration the conditions of anatomical normality and possible variants must be considered. Two examiners trained to use this software evaluated all the CBCT images using the method described. The kappa coefficient was used to assess interobserver agreement.

### Statistical analysis

Data were analyzed statistically by Fischer’s exact test to determine significant differences among the instrument groups on the walls. The significance level was set at 0.05 (SPSS v 20, Chicago, IL).

## Results

Tables 1 and 2 show the frequency of operative risk after RCP in the function of the different NiTi instruments assessed, using a specific algorithm and 3D reconstruction of the new CBCT software. There were no significant differences in operative risk in the comparative analysis between NiTi systems along distal or mesial walls of root canal thirds (*p* > 0.05).

In the cervical third, the frequency of mild operative risk after RCP with NiTi instruments capable of altering the spatial geometry in distal and mesial walls of mesiobuccal roots showed values above 87.5% and 100%, respectively. In the apical third, on both walls, no severe risk (0%) of altering the spatial geometry was found for any of these instruments tested. In mesiolingual roots, the frequency of mild risk of altering the spatial geometry after RCP with NiTi instruments in distal and mesial walls showed values above 80.9% and 100%, respectively. The instruments tested did not demonstrate severe risks of altering the spatial geometry after CPR in the apical thirds.

The Kappa results for interobserver agreement ranged from 0.86 to 0.96 for measurement in all root thirds on the CBCT images using e-Vol DX software.

**Table 1:** Frequency of operative risk after RCP in mesiobuccal roots with different NiTi engine-driven instruments capable of altering the spatial geometry, assessed using a specific algorithm and 3D reconstruction in a new CBCT software.

Operative risk	ProTaper Next		BioRace		Reciproc Blue		WaveOne Gold		P value	
Cervical third	Distal wall	Mesial wall	Distal wall	Mesial wall	Distal wall	Mesial wall	Distal wall	Mesial wall	Distal wall	Mesial wall
1. mild risk	19 (90.5%)	21 (100%)	18 (85.7%)	21 (100%)	20 (95.2%)	21 (100%)	19 (90.5%)	21 (100%)	0.866	1.000
2. moderate risk	2 (9.5%)	0 (0%)	2 (9.5%)	0 (0%)	1 (4.8%)	0 (0%)	2 (9.5%)	0 (0%)		
3. severe risk	0 (0%)	0 (0%)	1 (4.8%)	0 (0%)	0 (0%)	0 (0%)	0 (0%)	0 (0%)		
Middle third										
1. mild risk	12 (57.1%)	21 (100%)	18 (85.7%)	21 (100%)	16 (76.2%)	21 (100%)	18 (85.7%)	21 (100%)	0.124	1.000
2. moderate risk	9 (42.9%)	0 (0%)	3 (14.3%)	0 (0%)	5 (23.8 %)	0 (0%)	3 (14.3%)	0 (0%)		
3. severe risk	0 (0%)	0 (0%)	0 (0%)	0 (0%)	0 (0%)	0 (0%)	0 (0%)	0 (0%)		
Apical third										
1. mild risk	21 (100%)	21 (100%)	21 (100%)	21 (100%)	21 (100%)	21 (100%)	20 (95.2%)	21 (100%)	0.999	1.000
2. moderate risk	0 (0%)	0 (0%)	0 (0%)	0 (0%)	0 (0%)	0 (0%)	1 (4.8%)	0 (0%)		
3. severe risk	0 (0%)	0 (0%)	0 (0%)	0 (0%)	0 (0%)	0 (0%)	0 (0%)	0 (0%)		

**Table 2:** Frequency of operative risk after RCP in mesiolingual roots with different NiTi engine-driven instruments capable of altering the spatial geometry, assessed using specific algorithm and 3D reconstruction in a new CBCT software.

Operative risk	ProTaper Next		BioRace		Reciproc Blue		WaveOne Gold		P value	
Cervical third	Distal wall	Mesial wall	Distal wall	Mesial wall	Distal wall	Mesial wall	Distal wall	Mesial wall	Distal wall	Mesial wall
1. mild risk	20 (95.2%)	21 (100%)	17 (80.9%)	21 (100%)	18 (85.7%)	21 (100%)	18 (85.7%)	21 (100%)	0,667	1.000
2. moderate risk	1 (4.8%)	0 (0%)	4 (19.1%)	0 (0%)	3 (14.3%)	0 (0%)	3 (14.3%)	0 (0%)		
3. severe risk	0 (0%)	0 (0%)	0 (0%)	0 (0%)	0 (0%)	0 (0%)	0 (0%)	0 (0%)		
Middle third										
1. mild risk	14 (66.7%)	21 (100%)	12 (57.2%)	21 (100%)	17 (80.9%)	21 (100%)	17 (80.9%)	21 (100%)	0,309	1.000
2. moderate risk	7 (33.3%)	0 (0%)	7 (33.3%)	0 (0%)	4 (19.1%)	0 (0%)	4 (19.1%)	0 (0%)		
3. severe risk	0 (0%)	0 (0%)	2 (9.5%)	0 (0%)	0 (0%)	0 (0%)	0 (0%)	0 (0%)		
Apical third										
1. mild risk	21 (100%)	19 90.5%	21 (100%)	21 (100%)	18 (85.7%)	19 (90.5%)	19 (90.5%)	21 (100%)	0,095	0,323
2. moderate risk	0 (0%)	2 (9.5%)	0 (0%)	0 (0%)	3 (14.3%)	2 (9.5%)	1 (4.75%)	0 (0%)		
3. severe risk	0 (0%)	0 (0%)	0 (0%)	0 (0%)	0 (0%)	0 (0%)	1 (4.75%)	0 (0%)		

## Discussion

NiTi engine-driven instruments were safe regarding the operative risk of excessive removal of hard tissue from the root canal walls after preparation, regardless of the taper and kinematics of continuous rotation or reciprocal movement, based on the 3D spatial geometry method.

A variety of new NiTi systems have been made available incorporating specific characteristics such as alloys, designs, tapers, movements, torque, and rpm ^3^ ^,^ ^6^ ^,^ ^7^ ^,^ ^10^ ^,^ ^23^ ^,^ ^32^ ^,^ ^33^ ^,^ ^34^ ^,^ ^35^ ^,^ ^36^ ^,^ ^37^. A concern of instrument manufacturers has been the centering ability and canal transportation risk during RCP. Several studies have analyzed the behavior of NiTi instruments regarding these features ^21^ ^,^ ^22^ ^,^ ^32^ ^,^ ^33^ ^,^ ^34^ ^,^ ^35^ ^,^ ^36^. The ability to maintain the original shape of a canal after RCP requires further analysis. Permanent human teeth may have various root canal cross-sectional shapes (circular, conical-pyramidal, oval/long oval, flat/ribbon-like, eight-shaped, C-shaped, trapezoidal, drop-shaped, and other forms), as well as isthmuses, which must be duly ^25^. It should be noted that it may be difficult or impossible to maintain an unaltered original shape after the completion of an RCP. Finally, it is challenging to maintain centered eccentric shapes such as flat or oval canals, which are often present in human molars ^25^.

The present method aims to determine the spatial geometry of the prepared root canal in CBCT scans (MPR, multiplanar reformations, and 3D reconstructions). This concept can also assess the ability of endodontic instruments to create a centralized or decentralized root canal shape. Multidimensional visualization in 3D mode (with volumetric reconstruction or cinematic rendering), based on evaluation concerning the edges of the anatomical structure being measured, provides a new comparative parameter for the analysis of prepared root canals with different NiTi instruments.

In the present study, the standard reference for measurement in the CBCT scan was multiplanar reformations (MPR). In a recent study, the importance of CBCT cinematic rendering for clinical decision-making, teaching, and research in Endodontics was discussed ^30^. The most common way to post-process a CBCT scan volume is reformatting it in three planes (X, Y, and Z) for multiplanar reformations (MPR), allowing a professional to make multiple changes to the spatial position of these planes, thickness, brightness, contrast, and sharpness of the image. Another image post-processing method for CBCT scans uses an appropriate 3D model (volume rendering or cinematic rendering) that can support and promote a better understanding of the spatial geometry of RCP, as carried out in this study.

The analysis of spatial geometry in CBCT scans should be considered in association with dynamic navigation across the prepared pulp cavity, a feature that distinguishes the methodology used in the present study. Using this method, it is possible to identify the regions of operative risk (mild, moderate, and severe risk, based on the thickness of dentin-cement remaining) in all regions of the root canal (coronal, middle, and apical thirds), not only in the danger zones or the apical regions. After RCP it is common to find the pulp cavity positioned more directed towards the distal wall, compared to the mesial wall in coronal and middle thirds. The application of this geometric spatial analysis in clinical practice provides benefits for the correct planning and decision-making regarding the selection of instrument sequences and how much the root canal should be widened.

An interesting and widely used method was proposed by Gambil et al. ^21^ The centralization capacity was defined as the ability of the endodontic instrument to remain in the central axis of the root canal assessed by the values obtained in the measurement of distances for the calculation of the transport index using a General Electric 9800 Advantage Fast Scan CT unit. The extent and direction of canal transportation were measured starting with the shortest distance from the edge of the uninstrumented canal to the edge of the tooth in both mesial and distal directions and then comparing this with the same measurements taken from the instrumented images. The NiTi instruments (Mity file) used caused significantly less canal transportation, removed less volume of dentin, required less instrumentation time, and produced more centered and rounder canal preparations than K-flex stainless steel files. This method has been used in analysis using CBCT scans ^22^ ^,^ ^35^ ^,^ ^36^ and in MCT ^34^. In a previous study using CBCT scans ^22^, the apical transportation and centralization ability were not influenced by the type of mechanical movement and instruments used (oscillatory with K-FlexoFile, oscillatory with NiTiflex file, K3 rotary instrument, and RaCe rotary instrument) after RCP up to a #40.02 diameter in mesiobuccal of maxillary first molars. In another study ^33^, the volume of removed dentin, transportation, and centering ability of the ProTaper Next (PTN) system, with and without glide path preparation using CBCT scans, were compared at 3 mm, 5 mm, and 7 mm from the apex. The glide path/PTN instrumentation method presented better performance with fewer canal aberrations when compared with instrumentation performed with Path-File/PTN or PTN only. Haupt et al. ^34^ analyzed the RCP based on MCT of moderately curved mandibular molars using the reciprocating NiTi systems: S1 Plus Standard, WaveOne Gold Primary, and Reciproc R25. The following morphologic parameters were assessed: root canal volume and surface area, percentage of unshaped root canal walls, structure model, degree of centering ability, and canal transportation at 4 levels (1 mm, 3 mm, 5 mm, and 7 mm from the apex). RCP with the 3 NiTi systems did not result in significantly different dimensional changes, and there was no significant effect of the type of curvature on all tested parameters. The transportation and centering ability of RCP using different NiTi systems (ProTaper Next X4, Protaper Gold F4, Mtwo 40/.04, BioRaCe BR5, WaveOne Gold Large, and Reciproc R40) were also evaluated using CBCT images using Gambil et al. method ^21^ ^,^ ^35^. The analysis was carried out at 2 mm, 3 mm, and 4 mm from the apex, and 2 mm, 3 mm, and 4 mm below furcation. The results showed that all systems produced some root canal transportation. No file system achieved a perfect centering ability for RCP.

The results using the present method showed a limited operative risk after RCP, regardless of the degree of widening, the taper of the tested instruments, and the kinematics used. These data agree with the studies described above ^21^ ^,^ ^22^ ^,^ ^33^ ^,^ ^34^ ^,^ ^35^ ^,^ ^36^. The present method of root canal spatial geometry was established according to the interpretation of volume rendering (3D CBCT scans), which also used the parameters of measurements in MPR, followed by synchronization with volume rendering and operative risk analysis. A differential of this method is to identify via 3D volumetric reconstruction, the areas of operative risk along the walls of the root canal, both in axial sections and in the volume of the prepared pulp cavity. In addition, the relationship between the pulp cavity with the periodontal ligament and adjacent alveolar bone may be visualized with a specific algorithm (pulp cavity) for the CBCT e-Vol DX software which can suppress cementum/dentin structures and show the volume and shape of the prepared root canal (Figures 1 and 2B). 

The frequency of eccentric root canals (non-circular shapes and the anatomical variants observed) ^25^ ^,^ ^26^, in association with the characteristics of the designs and the taper of NiTi instruments, causes a tendency for natural action towards root areas of greater volume. This does not accurately distinguish instrument centering ability, as what is being assessed is the enlargement of the root canal lumen existing within a standard of convenience and the relationship between the canal anatomy and the instrument. The spatial geometry after RCP commonly differs from the original root shape, and this aspect is consistent with the problem that not all instruments will work equally on all root canal walls. In this context, instruments with particular features, such as TruNatomy, self-adjusting file, or XP-endo shaper, have been incorporated into the endodontic arsenal ^7^ ^,^ ^10^ ^,^ ^32^ to aid in the treatment of eccentric root canals, as well as anatomic variations. The biological aspects directed to the clinical results of the root canal treatment should constitute a focus of current understanding within a critical view of the behavior of NiTi instruments ^37^.

This CBCT software has contributed to tools for clinical application in health. Among some features, this software provides high-resolution images due to submillimeter voxel sizes, dynamic multi-plane imaging navigation, and the ability to change the volume parameters such as slice thickness and slice intervals and data correction by applying imaging algorithms and manipulating brightness and contrast ^2^ ^,^ ^26^ ^,^ ^28^ ^,^ ^30^. Other features make this CBCT software potentially applicable in clinical conditions such as blooming artifact reduction produced by high-density materials, such as metal intracanal posts ^27^ ^,^ ^38^. Additionally, slice thickness and sharpening may be customized, and its advanced noise reduction algorithm improves image quality. It also features filters for preset imaging and dedicated volume rendering, which may enlarge images over 1,000x in 3D reconstructions without loss of resolution. This photorealistic cinematic rendering tool, which uses Artificial Intelligence-related algorithms, has been developed for reconstructions ^2^ ^,^ ^30^.

Recently, Bueno and Estrela ^39^ proposed a computational modeling method for root canal endoscopy. This concept of communications and clinical operationalization based on the virtual visualization of the internal anatomy of root canals may impact the predictability of root canal treatments marking a new era in the metaverse of Endodontics.

## Conclusion

The spatial geometry method to assess operative risk allows clinical planning for a predictable enlargement of the root canal in all root thirds. Based on using a map-reading strategy with spatial geometry method on root canals in CBCT scans, NiTi engine-driven instruments did not present an increased operative risk during RCPs.
